# Three distinct atmospheric circulation patterns associated with high temperature extremes in South Korea

**DOI:** 10.1038/s41598-021-92368-9

**Published:** 2021-06-18

**Authors:** Han-Kyoung Kim, Byung-Kwon Moon, Maeng-Ki Kim, Jong-Yeon Park, Yu-Kyung Hyun

**Affiliations:** 1 Division of Science Education/Institute of Fusion Science, Jeonbuk National University, Jeonju, South Korea; 2 Department of Atmospheric Science, Kongju National University, Gongju, South Korea; 3 Department of Earth and Environmental Sciences, Jeonbuk National University, Jeonju, South Korea; 4 Operational Systems Development Department, National Institute of Meteorological Sciences, Seogwipo, South Korea

**Keywords:** Environmental sciences, Atmospheric science

## Abstract

The negative impact of extreme high-temperature days (EHDs) on people’s livelihood has increased over the past decades. Therefore, an improved understanding of the fundamental mechanisms of EHDs is imperative to mitigate this impact. Herein, we classify the large-scale atmospheric circulation patterns associated with EHDs that occurred in South Korea from 1982 to 2018 using a self-organizing map (SOM) and investigate the dynamic mechanism for each cluster pattern through composite analysis. A common feature of all SOM clusters is the positive geopotential height (GPH) anomaly over the Korean Peninsula, which provides favorable conditions for EHDs through adiabatic warming caused by anomalous downward motion. Results show that Cluster 1 (C1) is related to the eastward-propagating wave train in the mid-latitude Northern Hemisphere, while Cluster 2 (C2) and 3 (C3) are influenced by a northward-propagating wave train forced by enhanced convection in the subtropical western North Pacific (WNP). Compared to C2, C3 exhibits strong and eastward-extended enhanced convection over the subtropical WNP, which generates an anomalous high-pressure system over the southern part of the Kamchatka Peninsula, reinforcing EHDs via atmospheric blocking. Our results can contribute to the understanding of East Asia climate variability because wave trains influence the climate dynamics of this region.

## Introduction

An extreme high-temperature day (EHD) requires attention because it can have serious negative effects on all aspects of human life^1^. Increasing greenhouse gas concentrations have led to a rise in global air temperatures over the past decades^2–5^. Along with an increase in the air temperature, EHDs have also occurred more frequently, persistently, and intensely over this period^6–8^.

In the summer of 2010, western Russia suffered from unprecedented EHDs, more than those recorded since EHD recording commenced in the nineteenth century^9,10^. A record-breaking number of EHDs and droughts occurred in East Asia, causing more than 3,000 deaths in South Korea in 1994^11^. However, it is important to recognize that recent severe EHDs cannot be explained solely by the thermodynamic effects of global warming^12–14^.

In addition to the thermodynamic effects of global warming, studies have revealed that regional processes (e.g., foehn winds) and dynamic effects (e.g., large-scale teleconnection patterns and atmospheric blocking) as key factors influencing the generation of EHDs^15–17^. For example, foehn winds can significantly increase the surface air temperature through thermodynamic processes over the downslope region of a mountain^15^.

Many previous studies have emphasized the importance of the dynamic effects that induce EHDs^5,12,14,18–22^. Dynamically, a persistently positive geopotential height (GPH) anomaly is accepted as the most important atmospheric factor leading to the occurrence of EHDs because it provides favorable conditions for EHDs via adiabatic warming caused by downward motion^20,22,23^. Focusing on the Korean Peninsula, EHD-related atmospheric circulation patterns are intimately associated with two types of wave trains generated by enhanced convection over the subtropical western North Pacific (WNP)^5,14,18,20,22^ and mid-latitude Northern Hemisphere wave dynamics^14,19,20–22^. For instance, enhanced convection over the South China Sea acts as a source of the northward-propagating wave train, which resembles the Pacific-Japan (PJ) teleconnection pattern^24^. This wave train generates a positive GPH anomaly over the Korean Peninsula, leading to frequent EHDs^18^. In a similar vein, a previous study^5^ investigated the record-breaking EHDs over South Korea in 2016. It was argued that strong enhanced convection over the western to central subtropical Pacific in August 2016 induced the northeastward-propagating wave train, which led to the development of a positive GPH anomaly over the Kamchatka Peninsula. This anomaly led to atmospheric blocking in the downstream region of the Korean Peninsula. In addition, an anomalously high GPH and surface temperature were observed in Mongolia in August 2016. The corresponding northerly wind caused warm advection from Mongolia into South Korea, leading to severe EHDs in August 2016.

A recent study^21^ identified the roles of Scandinavian^25^ and circumglobal teleconnection (CGT)^26^ patterns, which may be considered eastward-propagating wave trains, in the development of EHDs over East Asia, including the Korean Peninsula. They showed that East Asian EHDs were closely related to the CGT pattern before the mid-1990s, whereas the Scandinavian pattern was crucial in explaining recent EHDs over East Asia. These large-scale teleconnection patterns generate cyclonic and anticyclonic circulations along the wave path, thus modulating the EHDs over East Asia.

To understand the independent roles of the above-mentioned wave trains in the occurrence of EHDs over South Korea, the EHD-related large-scale atmospheric circulation patterns were heuristically classified into two distinct types of wave trains, i.e., zonal and meridional, based on their spatial distribution characteristics using composite analysis^20^. The results showed that meridional wave type EHDs tend to occur more frequently in recent years, whereas zonal wave type EHDs dominated during the 1990s.

**Figure 1:**
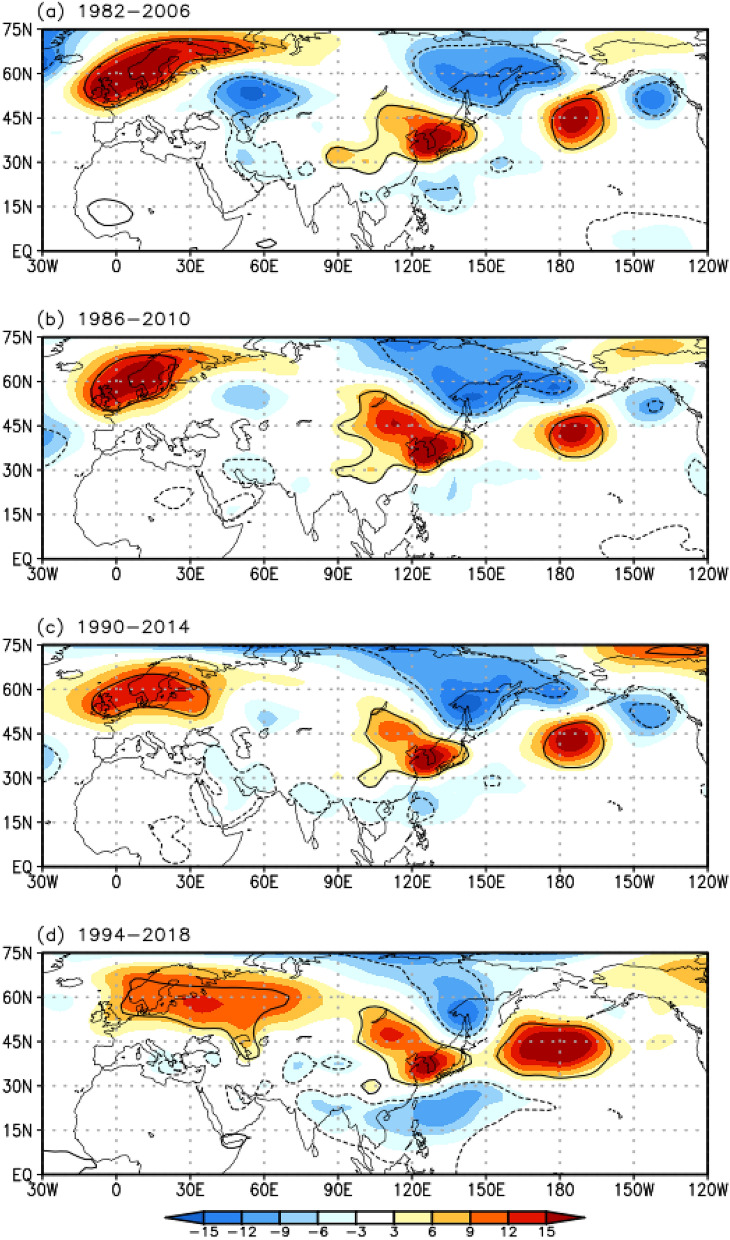
Composite maps of (**a**) the 850-hPa GPH (shaded; m) anomalies for the selected EHDs in South Korea from 1982–2006. (**b**), (**c**), and (**d**) are the same as (**a**) but for 1986–2010, 1990–2014, and 1994–2018, respectively. Contours represent statistically significant areas satisfying the 99% confidence level based on the Student’s *t* test. Maps were generated using GrADS version 2.1.a1 (http://cola.gmu.edu/grads/downloads.php).

An analysis of the 25-year sliding composite maps of the 850-hPa GPH anomalies for the EHDs in South Korea shows that the eastward-propagating wave train gradually diminishes when the 850-hPa GPH anomalies are calculated for the 25-year sliding periods (Fig. 1a–d). In contrast, the northward-propagating wave train makes a distinct appearance during recent periods (Fig. 1d), as compared with the past (Fig. 1a–c). These observations broadly coincide with the results of a previous study^20^. Furthermore, considering the spatial features of the two types of wave trains, the diminishing eastward-propagating wave train, as time progresses, can be interpreted as the canceling out of the significant signal from various large-scale factors. For example, the negative GPH anomaly component of a wave train passing through a specific region, where a persistent positive GPH anomaly is located, can cause extinction of the significant signal. To synthetically understand these spatial and temporal variations, clustering analysis can be a viable alternative tool. Recently, the long-term relationship between South Korean heat waves and the summer North Atlantic oscillation (NAO) was examined^27^ via the *K*-means clustering method. This study showed that the number of heat wave days related to the positive (negative) GPH anomaly over the Kamchatka Peninsula increased (decreased) significantly from 2000 to 2018 compared with that from 1981 to 1999. This long-term change in the GPH anomaly was due to the phase transition of the summer NAO from positive to negative. However, the 500-hPa GPH anomalies over East Asia and the North Pacific (i.e., 0°–70° N, 80°–230° E) were used as input for clustering analysis, even though the EHDs in South Korea are influenced by the eastward-propagating wave train across the Eurasian continent^14,19,20–22^. Furthermore, the northward-propagating wave train, forced by enhanced convection over the WNP^5,14,18,20,22,24^, which is a major mechanism associated with the EHDs in South Korea, is not captured in their clustering results (Fig. 1 in Ref.^27^). The random selection of the initial centroids causes the *K*-mean clustering method to converge to the local optimum solution and become sensitive to outliers in the data. Therefore, in this study, we use a self-organizing map (SOM), which is an unsupervised artificial neural network, to classify the large-scale atmospheric circulation patterns associated with the EHDs in South Korea. The key strength of the SOM is that it concisely describes the clustered patterns of variability, including nonlinearity in the data^28^. Thus, this method has already been extensively applied to extract the independent patterns of the East Asian summer monsoon^28^, Indian monsoon intraseasonal oscillation^29^, El Niño Southern oscillation^30–32^, and tropical cyclone tracks over the WNP^33^. Furthermore, to select the cluster number for the atmospheric circulation patterns that lead to EHDs in South Korea, we use a statistically distinguishable test, known as a false discovery rate (FDR). After performing clustering with the SOM, we investigate the spatial and temporal variability, as well as the dynamic mechanism regulating EHDs in South Korea, for each cluster pattern.

## Characteristics of atmospheric circulation patterns related to EHDs in South Korea

Figure 1a shows the composite map of the 850-hPa GPH anomalies for the selected EHDs over South Korea in June–July–August (JJA) from 1982 to 2006. A significant positive 850-hPa GPH anomaly notably appears over the Korean Peninsula, which is the key factor for the occurrence of EHDs, as stated in the introduction.

A notable feature of the EHD-related atmospheric circulation patterns is the eastward-propagating wave train in the mid-latitudes of the Northern Hemisphere, from northern Europe to East Asia across the Eurasian continent, which is positive in northern Europe and East Asia and negative in the Ural Mountains (Fig. 1a). In addition, the north–south dipolar structure of the 850-hPa GPH anomaly, which is characterized by a positive over East Asia and negative over the subtropical WNP, is also a prominent feature in the EHD-related atmospheric circulation patterns (Fig. 1a). This dipolar structure closely resembles the northward-propagating wave train, which is known as the PJ teleconnection pattern^24^. The composite map implies that EHDs in South Korea are highly correlated with the two types of wave trains^20,22^. However, the significant factor is that the EHD-related atmospheric circulation patterns vary based on a reference period for the composite map. To obtain more detail on the changes in the EHD-related atmospheric circulation patterns, we examined the composite maps of the 850-hPa GPH anomalies for the selected EHDs in South Korea during each of the four overlapping 25-year periods (i.e., 1982–2006, 1986–2010, 1990–2014, and 1994–2018). Figure 1a–d indicates that the eastward-propagating wave train gradually fades away when the 850-hPa GPH anomalies are calculated for the 25-year sliding periods, especially with the weakening negative GPH anomaly over the Ural Mountains. In contrast, the northward-propagating wave train is more well-organized in the recent period (1994–2018; Fig. 1d) than during previous periods (1982–2006, 1986–2010, and 1990–2014; Fig. 1a–c). These changes imply the following two features. (1) The dominant mechanism for EHDs in South Korea can change over a long-term timescale, e.g., the eastward- and northward-propagating wave trains before and after a specific period, respectively, dominantly influence the EHDs in South Korea. (2) Different large-scale atmospheric circulation patterns that induce EHDs in South Korea can cancel out the GPH anomaly over a specific region; in other words, if negative and positive GPH anomalies from individual samples are superimposed on a composite map, the significant signal can be rendered extinct. To interpret these changes, we classified the atmospheric circulation patterns relevant to the EHDs in South Korea using the SOM, where the cluster number was statistically selected by the FDR.

## Maximum cluster number of distinguishable SOM cluster patterns

To determine the cluster number for the atmospheric circulation patterns that lead to EHDs in South Korea, we executed the SOM by changing the number of clusters from two to eight, and then applied the FDR to the clustering results. Supplementary Fig. S1 shows the outcomes of the FDR, which display the number of statistically indistinguishable SOM cluster pairs as a function of the number of clusters. When the number of clusters is increased from two to three, all cluster pairs are statistically distinguishable. In contrast, when the number of clusters is equal to or greater than four, the number of indistinguishable cluster pairs exceeds zero. Therefore, the maximum cluster number that is statistically distinguishable was set to three.

**Figure 2:**
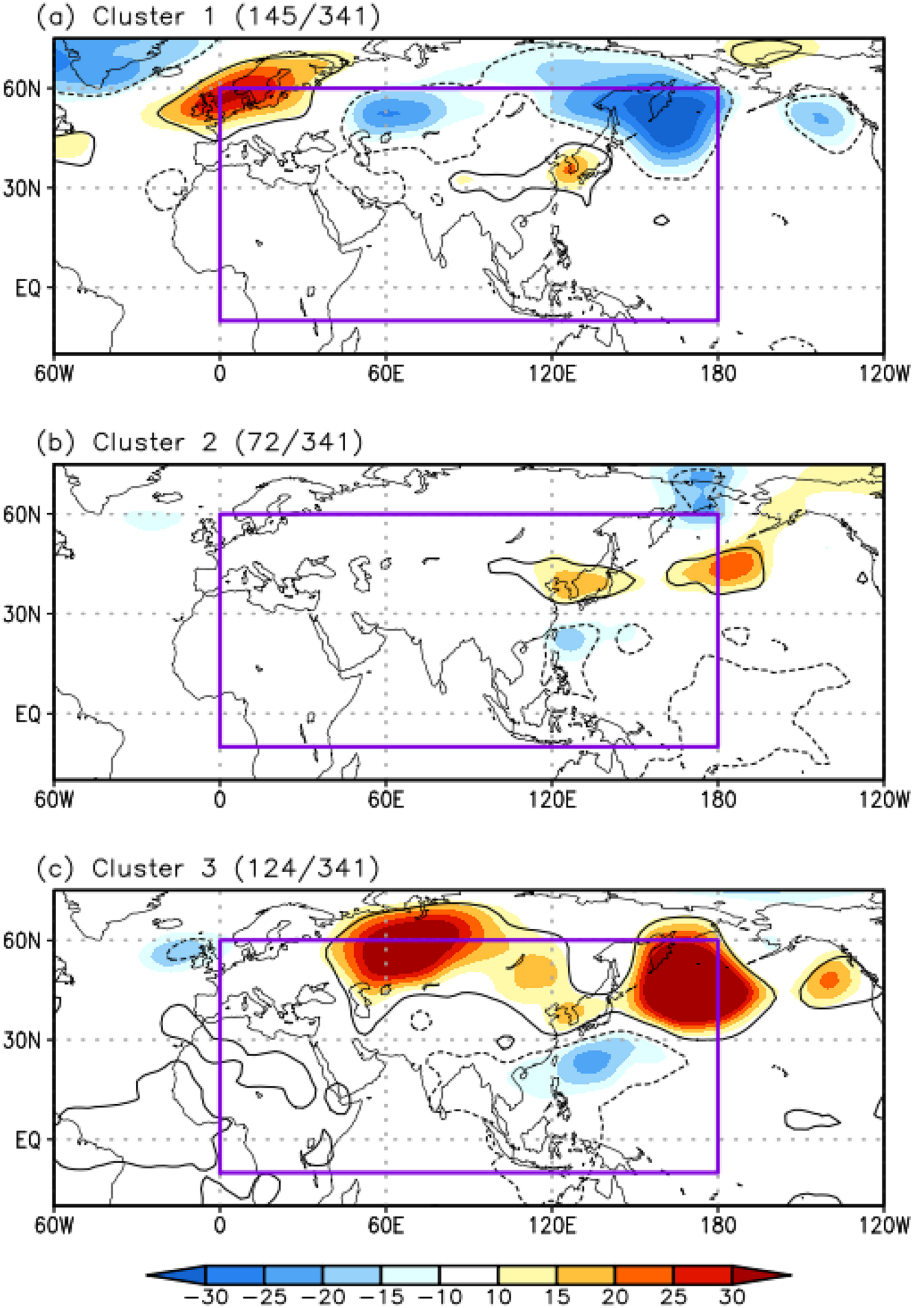
Composite maps of the 850-hPa GPH (shaded; m) anomalies for the three SOM cluster patterns. The purple box (i.e., 0°–180° E and 10° S–60° N) denotes the region of the input vector for the SOM. Contours represent statistically significant areas satisfying the 99% confidence level based on the Student’s *t*-test. The number of EHDs in each cluster are shown in parentheses. Maps were generated using GrADS version 2.1.a1 (http://cola.gmu.edu/grads/downloads.php).

**Figure 3:**
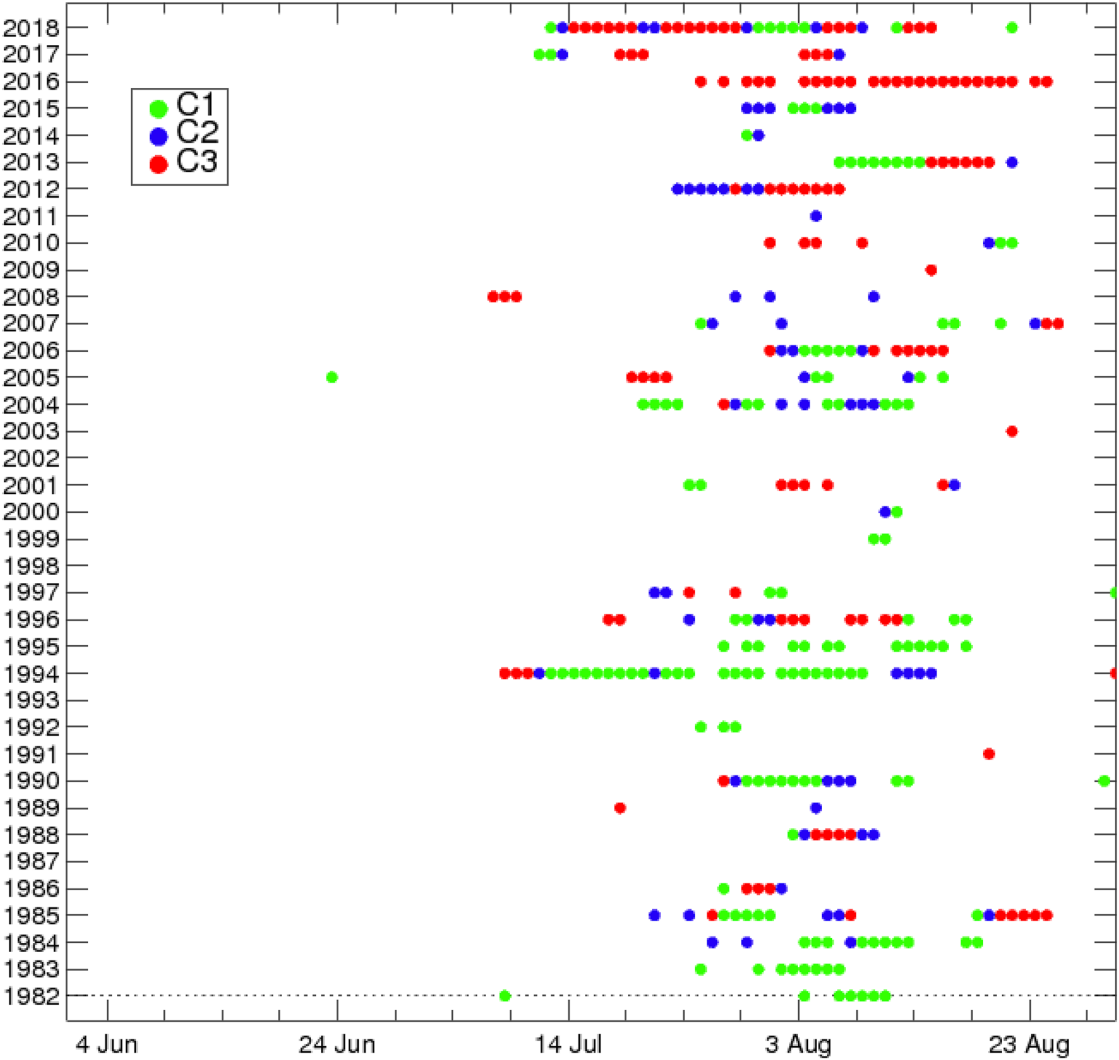
Daily temporal distributions of the three SOM cluster patterns.

Figure 2 shows the composite maps of the 850-hPa GPH anomalies for the three SOM cluster patterns. A common feature of all of the SOM cluster patterns is the positive 850-hPa GPH anomaly over East Asia, including the Korean Peninsula, which is the most predominant pattern generating EHDs in South Korea. In contrast, each cluster has a different large-scale atmospheric circulation pattern, especially over the subtropics and mid-latitudes of the Northern Hemisphere. Figure 3 illustrates the daily temporal distributions of the three SOM cluster patterns. Most EHDs of each cluster occur consecutively, which reflects their synoptic-scale characteristic. The detailed spatiotemporal features and dynamic mechanism of each cluster pattern were investigated, as described below.

## Clustering results

### Spatiotemporal variability and dynamic mechanism of Cluster 1

Cluster 1 (C1) accounts for 42.5% (145/341) of all EHDs and is the most frequent among all cluster patterns. C1 exhibits the strongest mean intensity (32.72 ℃; Table 1) and highest standard deviation (0.96 ℃; Table 1) in the daily maximum temperatures.

**Table 1:** Mean intensity and standard deviation for the three SOM cluster patterns.

Cluster number	C1	C2	C3	Total
Intensity (℃)	32.72	32.50	32.64	32.64
Standard deviation (℃)	0.96	0.75	0.83	0.87

The low-level atmospheric circulation pattern of C1 fluctuates mainly in the mid-latitudes of the Northern Hemisphere (Fig. 2a). This fluctuation can be identified by the zonally elongated 850-hPa GPH anomaly patterns, which are positive in northern Europe and the Korean Peninsula and negative in the Ural Mountains. We note that these anomaly patterns are closely analogous to the eastward-propagating wave train^22^ (Figs. 1a, 2a), as well as to the zonal wavelike pattern^20^. As stated above, the eastward-propagating wave train appears to be more closely related to EHDs over South Korea in the past than in recent periods (Fig. 1). To confirm this, we calculated the temporal distribution of the frequency in C1, which indicates that the high-frequency years for C1 are concentrated before the late 1990s (green circle in Fig. 3). In particular, in 1994, which is one of the years with severe EHDs in South Korea, the frequency of C1 was higher than the frequencies of the other clusters (green circle in Fig. 3), which is in good agreement with the results of a previous study^20^.

**Figure 4:**
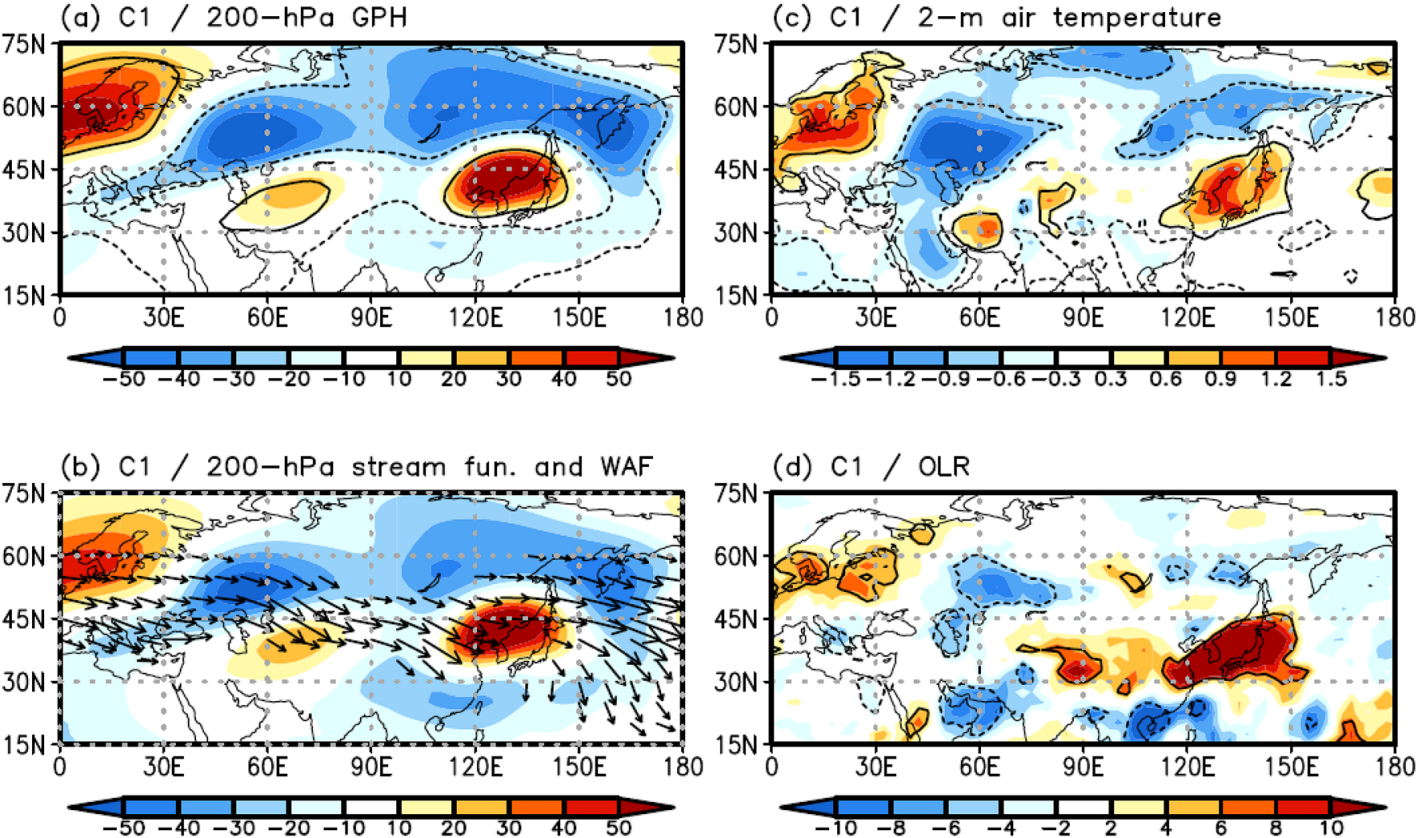
Composite map of (**a**) the 200-hPa GPH (shaded; m) anomalies for C1. (**b**), (**c**), and (**d**) are the same as (**a**) but for the 200-hPa stream function (shaded; m^2^ s^–1^) and WAF (vector; m^2^ s^–2^), 2-m air temperature (shaded; ℃), and OLR (shaded; W m^–2^) anomalies, respectively. WAF was omitted when its magnitude was less than 15 m^2^ s^–2^. Contours represent statistically significant areas satisfying the 99% confidence level based on the Student’s *t*-test. Maps were generated using GrADS version 2.1.a1 (http://cola.gmu.edu/grads/downloads.php).

To explore the modulating mechanism of this cluster on EHDs in South Korea, the atmospheric variables were investigated. Figure 4a shows the composite map of the 200-hPa GPH anomalies for the EHDs of C1. The 200-hPa GPH anomaly patterns largely agree with the 850-hPa GPH anomaly patterns from northern Europe to East Asia (Figs. 2a, 4a). The GPH anomaly at both levels, exhibiting the same characteristics, signifies a barotropic structure, which implies a close relationship between C1 and the barotropic wave train in the mid-latitudes of the Northern Hemisphere. Based on the analysis of the 200-hPa wave activity flux (WAF)^34^ and stream function, we can confirm that the wave train propagates eastward over northern Europe (0°–30° E, 50°–70° N), with a positive stream function anomaly, and then propagates southeastward in the Ural Mountains (40°–80° E, 40°–60° N), with a negative stream function anomaly (Fig. 4b). The direction of the wave train persists up to the Korean Peninsula (115°–140° E, 35°–45° N), with a positive stream function anomaly (Fig. 4b). Consequently, a positive barotropic GPH anomaly develops over the Korean Peninsula owing to this wave train. An anomalously high 2-m air temperature and suppressed convection are then induced by downward motion (Fig. 4c, d), which results in frequent EHDs in South Korea.

### Spatiotemporal variability and dynamic mechanism of Cluster 2

Only 21.1% (72/341) of all EHDs appear in Cluster 2 (C2), which is the least frequent pattern among the clusters (blue circle in Fig. 3). Moreover, this cluster has the weakest intensity (32.50 ℃; Table 1) and lowest standard deviation (0.75 ℃; Table 1).

The main spatial feature of C2 is a north–south dipole structure in the 850-hPa GPH anomaly, which is positive over the Korean Peninsula and negative over the subtropical WNP (Fig. 2b). This structure highly correlates with a northward-propagating wave train, such as the widely known PJ teleconnection pattern^5,14,18,22,24^ and meridional wavelike pattern^20^. Contrarily, there is no statistically significant circulation anomaly over the Eurasian continent (Fig. 2b). These spatial features denote that a northward-propagating wave train influences C2.

**Figure 5:**
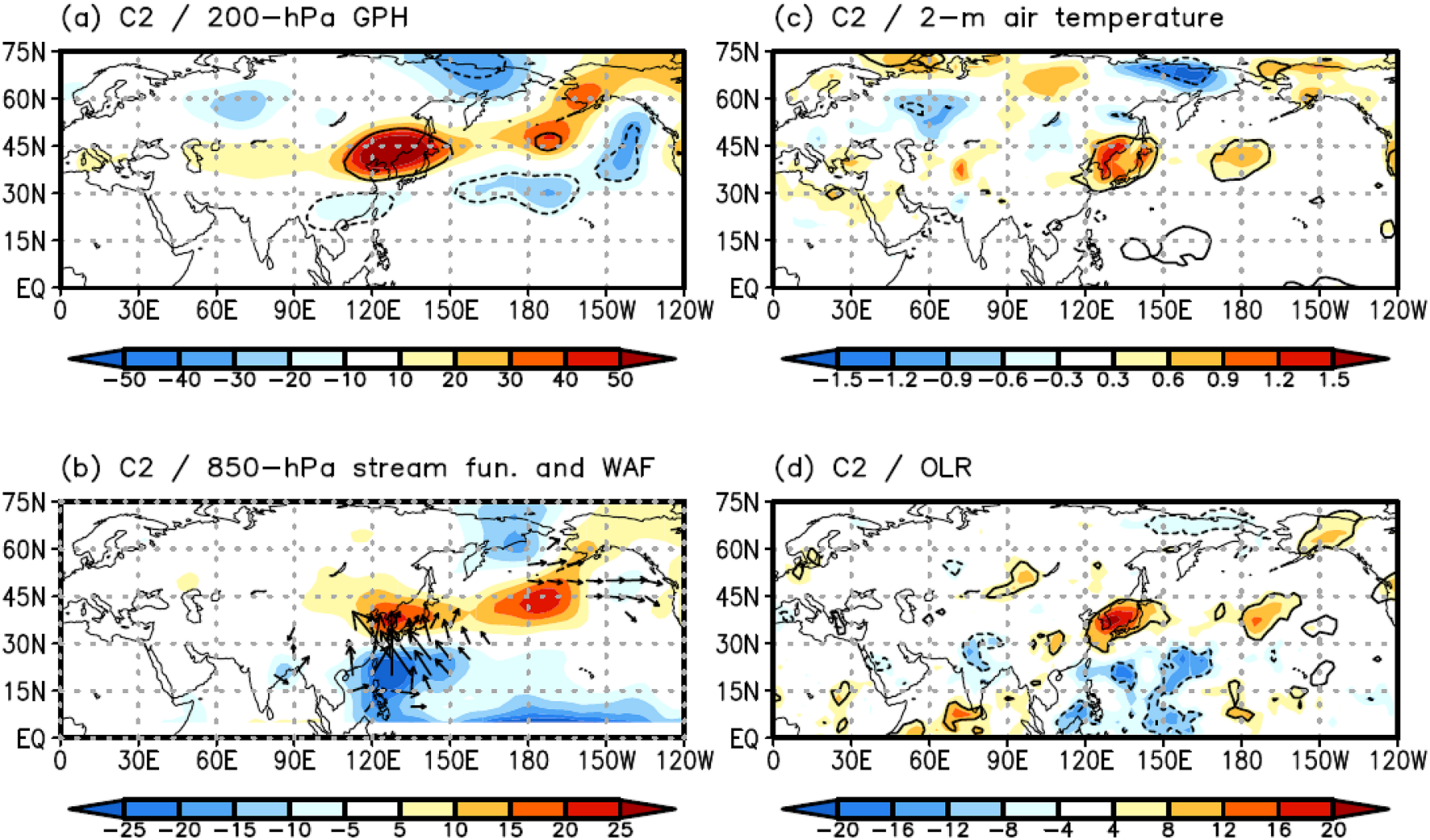
Composite map of (**a**) the 200-hPa GPH (shaded; m) anomalies for C2. (**b**), (**c**), and (**d**) are the same as (**a**) but for the 850-hPa stream function (shaded; m^2^ s^–1^) and WAF (vector; m^2^ s^–2^), 2-m air temperature (shaded; ℃), and OLR (shaded; W m^–2^) anomalies, respectively. WAF was omitted when its magnitude was less than 15 m^2^ s^–2^. Contours represent statistically significant areas satisfying the 99% confidence level based on the Student’s *t* test. Maps were generated using GrADS version 2.1.a1 (http://cola.gmu.edu/grads/downloads.php).

Figure 5a–d illustrates the composite maps of the 200-hPa GPH, 850-hPa WAF/stream function, 2-m air temperature, and OLR anomalies for C2, respectively. A positive barotropic structure in the GPH anomaly can be observed over the Korean Peninsula (Figs. 2b, 5a), which leads to a significant high-temperature anomaly (Fig. 5c) and suppressed convection due to downward motion (Fig. 5d). We note that a significant negative 850-hPa GPH anomaly can be found over the subtropical WNP (Fig. 2b), whereas there is no significant 200-hPa GPH anomaly over that area (Fig. 5a). These facts imply that the significant low-level circulation anomaly over the subtropical WNP may be due to convective heating. To investigate this, we examined the composite map of the OLR anomaly for the EHDs in C2. The results indicate significantly enhanced convection over the subtropical WNP, generating low-level cyclonic circulation as a Gill-type response^35^ (Figs. 2b, 5d). The convective anomaly activates the northward-propagating wave train^36,37^, inducing the positive barotropic structure of the GPH anomaly over East Asia, including the Korean Peninsula. The northward-propagating wave train related to C2 can also be confirmed when using the 850-hPa WAF and stream function (Fig. 5b). The WAF originates from the subtropical WNP with a negative stream function and arrives in the Korean Peninsula with a positive stream function. This result supports the northward-propagating wave train as a crucial factor modulating the EHDs in C2.

### Spatiotemporal variability and dynamic mechanism of Cluster 3

Cluster 3 (C3) is the second most frequent cluster pattern, accounting for 36.4% (124/341) of the total EHDs. It is significant that the high-frequency years of C3 occur in the recent period, particularly in 2016 and 2018 (red circle in Fig. 3a). This cluster exhibits a moderate mean intensity and standard deviation (32.64 ℃ and 0.83 ℃, respectively; Table 1).

**Figure 6:**
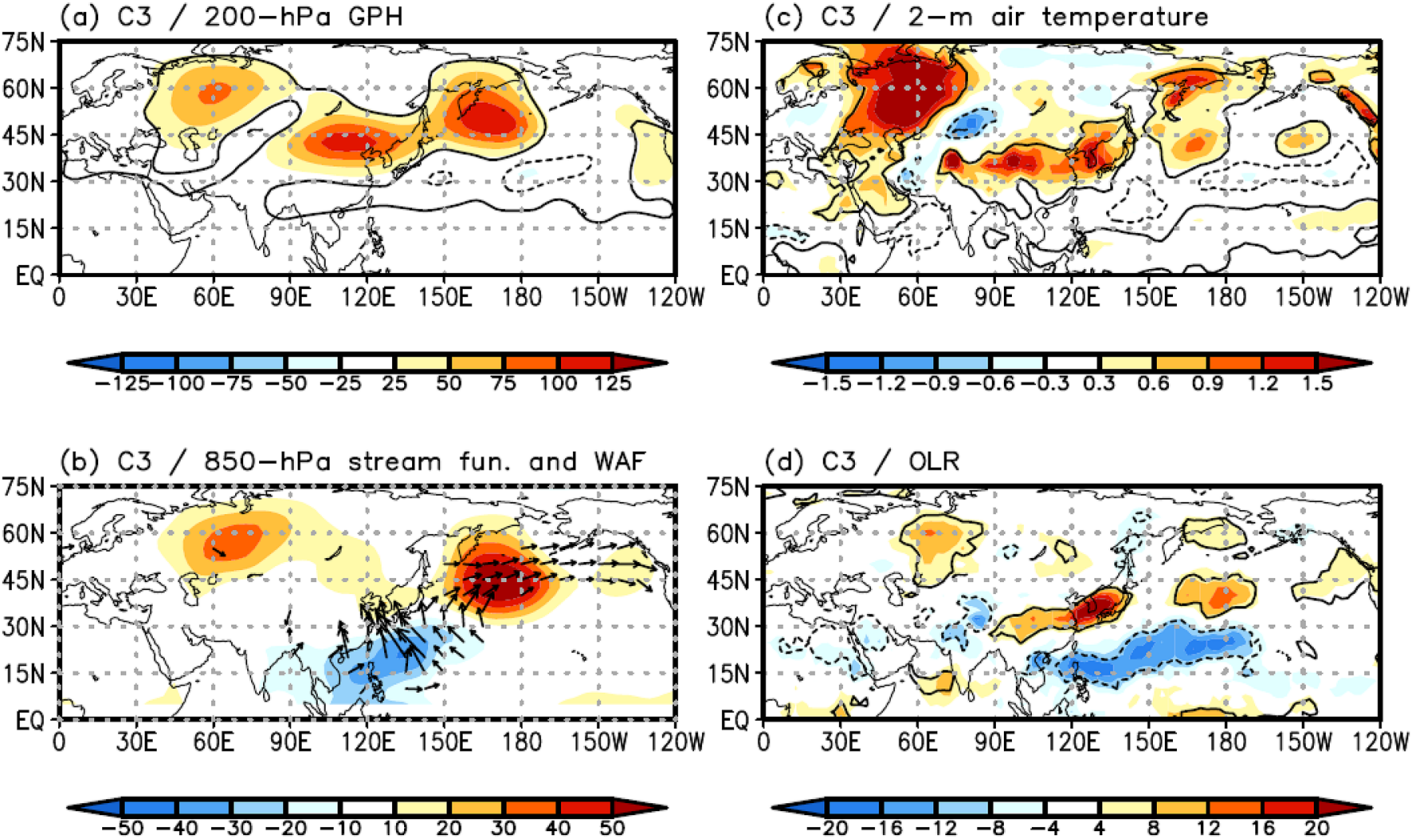
Composite map of (**a**) the 200-hPa GPH (shaded; m) anomalies for C3. (**b**), (**c**), and (**d**) are the same as (a) but for the 850-hPa stream function (shaded; m^2^ s^–1^) and WAF (vector; m^2^ s^–2^), 2-m air temperature (shaded; ℃), and OLR (shaded; W m^–2^) anomalies, respectively. WAF was omitted when its magnitude was less than 15 m^2^ s^–2^. Contours represent statistically significant areas satisfying the 99% confidence level based on the Student’s *t* test. Maps were generated using GrADS version 2.1.a1 (http://cola.gmu.edu/grads/downloads.php).

A spatial feature of C3 is that the positive 850-hPa GPH anomalies extend widely over the mid-latitude regions of the Ural Mountains, East Asia, and southern part of the Kamchatka Peninsula, forming a “V” shape (Fig. 2c). This spatial feature is also observed in the 200-hPa GPH anomalies (Fig. 6a), implying a barotropic structure in the GPH anomalies. The negative 850-hPa GPH anomaly over the subtropical WNP is also a significant feature of C3 (Fig. 2c), which is similar to the anomaly pattern of C2; however, the center of the anomaly in C3 is shifted eastward, and its intensity is stronger compared to that of C2 (Fig. 2b, c). The differences in the negative 850-hPa GPH anomaly over the subtropical WNP between the two clusters may be due to the different structures in the enhanced convection. To examine this, we investigated the composite map of the OLR anomaly for the EHDs in C3. The results indicate a zonally elongated enhanced convection from the western to central North Pacific (Fig. 6d), which extends further eastward and is stronger than that of C2, producing the eastward-extended and stronger negative low-level GPH anomaly as a Gill-type response^35^ (Fig. 2c).

Similar to that of C2, the enhanced convection over the subtropical WNP of C3 can generate the northward-propagating wave train^5,14,18,20,22,24^. However, based on the different properties of the enhanced convection, we would expect that the wave train is distinguishable from the wave train in C2. To investigate the propagation of the wave train, we analyzed the composite map of the 850-hPa WAF and stream function for C3 (Fig. 6b). The results show that the wave train is initiated over the subtropical WNP with a negative stream function, resembling C2 (Fig. 6b). However, the wave propagates toward the Korean Peninsula and southern part of the Kamchatka peninsula, leading to the development of positive GPH structures in both regions, with a positive stream function (Fig. 6b). The positive GPH anomaly over the Korean Peninsula leads to an anomalous increase in the high 2-m air temperature (Fig. 6c) and suppressed convection (Fig. 6d). In addition, the high-pressure system over the southern part of the Kamchatka Peninsula yields atmospheric blocking in the downstream region of the Korean Peninsula, which may interrupt the eastward migration of the anticyclone anomaly over the Korean Peninsula, increasing the number of EHDs in South Korea^5^ (Figs. 2c, 6a).

The positive GPH anomaly over the Ural Mountains can be observed in the atmospheric circulation pattern of C3 (Figs. 2c, 6a). However, this anomaly is not related to the northward-propagating wave train activated by enhanced convection over the WNP (Fig. 6b) and is unlikely to contribute to the eastward-propagating wave train toward the Korean Peninsula through the upper level WAF (Supplementary Fig. S2). We speculate that this positive GPH anomaly may be reflected by a persistent high-pressure system on a long-term timescale rather than on a synoptic timescale. To examine this speculation, an empirical orthogonal function (EOF) analysis was applied to the JJA mean 850-hPa GPH anomaly over the Eurasian continent from 1982–2018 to extract the dominant patterns of interannual variability in the summertime 850-hPa GPH anomaly over the Eurasian continent. The spatial pattern of the first EOF mode closely parallels the Scandinavian pattern^25^ (Supplementary Fig. S3a). Moreover, the first PC strongly correlates with the Scandinavian pattern index (*r* = 0.61), which can be obtained from the Climate Prediction Center of the National Oceanic and Atmospheric Administration (NOAA) (https://www.cpc.ncep.noaa.gov/data/teledoc/scand.shtml).

The second EOF mode is characterized by the positive GPH anomaly over the Ural Mountains (Supplementary Fig. S3b), which strongly resembles the positive GPH anomaly over the Ural Mountains in the atmospheric circulation pattern of C3 (Fig. 2c). It is important that the dominant phase of the normalized second PC has changed from negative to positive since 2009 based on the Rodionov regime shift algorithm^38^, with a cut-off length of 10 years and a 90% confidence level (Supplementary Fig. S4). Thus, the entire analysis period can be divided into two sub-periods based on the dominant second EOF mode: the negative (i.e., 1982–2008, P1) and positive (2009–2018, P2) sub-periods. As the high-frequency year of C3 appears more frequently in P2 than P1 (red circle in Fig. 3), the anomalous high GPH anomaly over the Ural Mountains in the C3 pattern may result from the reflected dominant positive phase of the second EOF mode. To further investigate the change in the positive GPH anomaly over the Ural Mountains in the C3 pattern on a long-term timescale, we explored the composite maps of the 850-hPa GPH anomaly in C3 for periods P1 and P2 (Supplementary Fig. S5). The results show that the northward- and northeastward-propagating wave trains generated by enhanced convection over the WNP, which are the main mechanisms that induce EHDs in C3, are apparent in both periods (Supplementary Fig. S5). Nevertheless, the positive GPH anomaly over the Ural Mountains in P2 is stronger than that in P1, supporting the inference of a long-term variation in the positive GPH anomaly caused by the second EOF mode. The long-term variation in the positive GPH anomaly over the Ural Mountains appears to stem from a significant decline in the soil moisture in that region (not shown). However, the observation data are insufficient to confirm this relationship; this should be examined further using an Earth system climate model.

## Results of linear baroclinic model experiments

**Figure 7:**
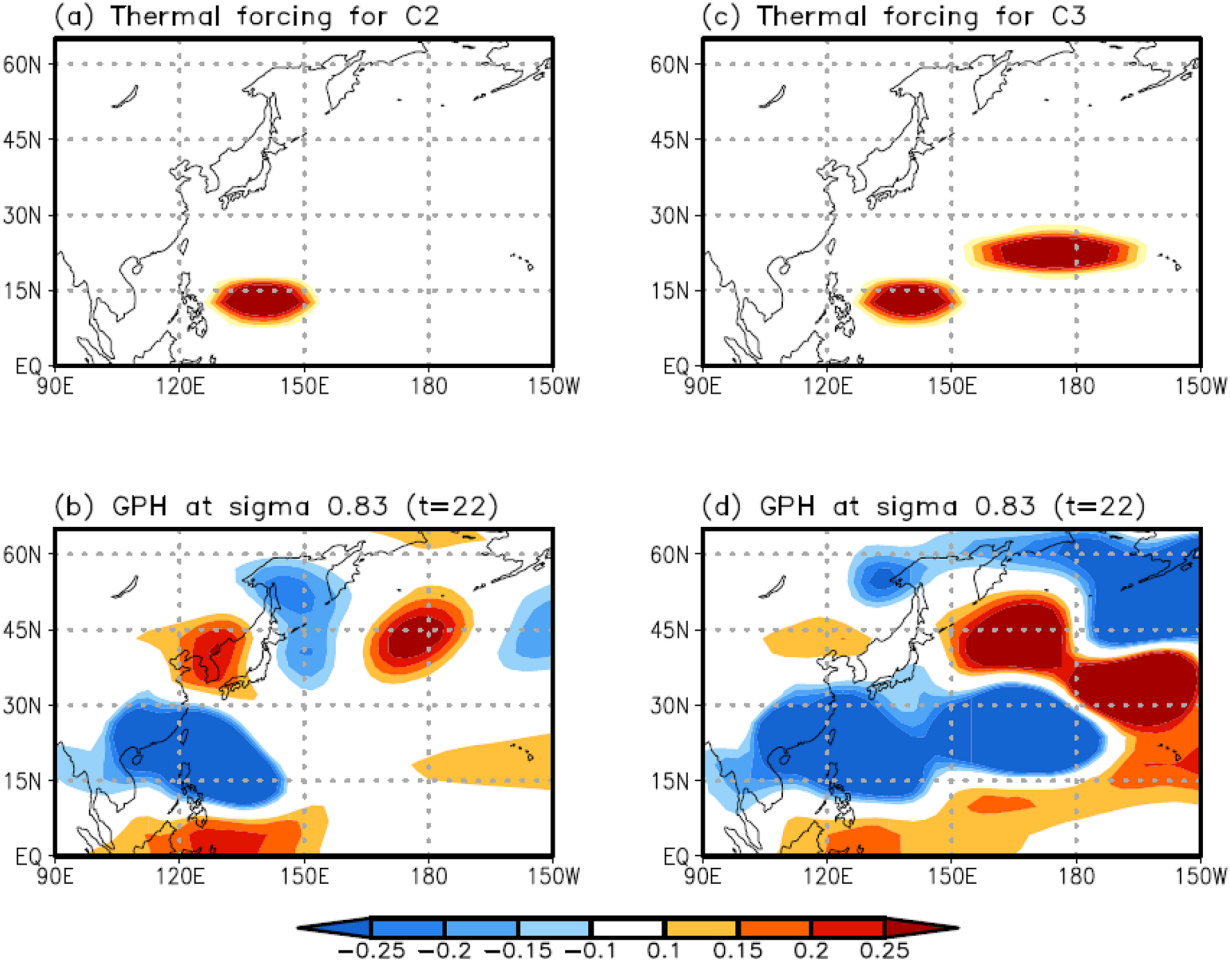
Horizontal distribution of the prescribed heating (K d^–1^) using the LBM experiment for (**a**) C2 and (**c**) C3. Maximal heating was set at a sigma level of 0.45. (**b**) and (**d**) show the 22-day integration results for the response of the 0.83 sigma level GPH anomaly fields to the prescribed heating for (**a**) and (**c**), respectively. Maps were generated using GrADS version 2.1.a1 (http://cola.gmu.edu/grads/downloads.php).

Linear baroclinic model (LBM) experiments were conducted to understand the impact of different forms of enhanced convection over the subtropical WNP on northward- and northeastward-propagating wave trains. Thermal heating represents the enhanced convection in C2. Vertically, the heating has a sinusoidal profile, with a maximum of 1 K day^–1^ at a sigma level of 0.45. Horizontally, the heating has a cosine-squared profile in an elliptical region, with a single center at 13°N and 140°E, where the radii of the region in the latitudinal and longitudinal directions are 5° and 15°, respectively (Fig. 7a). For this experiment, the JJA mean climatological (i.e., 1982–2018) variables were considered as the basic state because the frequency of C2 is approximately uniform during the entire analysis period (blue in Fig. 3a).

The LBM experiment was integrated for 30 days, and the response of the atmospheric circulation to the prescribed heating approached a steady state after day 20. After the integration of this model, we investigated the low-level (i.e., 0.83 sigma level) GPH anomaly. Consequently, the result on day 22 shows a negative GPH anomaly over the subtropical WNP, which results from a Gill-type response to the prescribed heating, whereas a positive low-level GPH anomaly is evident over the Korean Peninsula (Fig. 7b). The north–south dipolar structure in the GPH anomaly is similar to the atmospheric circulation pattern of C2 (Fig. 2b), confirming that the enhanced convection over the subtropical WNP can be a heat source that induces a northward-propagating wave train, including the enhanced GPH over the Korean Peninsula.

Thermal heating that can represent the enhanced convection for C3 has the same vertical structure as C2. However, the horizontal structure of this heating has a cosine-squared profile in two elliptical regions with double centers at 13°N, 140°E and 22.5°N, 180°E. The radii of the first and second regions in the latitudinal and longitudinal directions are 5° and 15° and 5° and 25°, respectively (Fig. 7c). In this experiment, the basic state corresponded to the 2009–2018 JJA mean variables because C3 is dominant during and after 2009 (red circle in Fig. 3).

The result on day 22 exhibits a more eastward-extended and stronger negative low-level GPH anomaly over the subtropical WNP than the previous experiment, which also results from a Gill-type response to the prescribed heating (Fig. 7d). The prescribed heating generates the northward- and northeastward-propagating wave train, developing the positive low-level GPH anomaly over the Korean Peninsula and southern part of the Kamchatka Peninsula (Fig. 7d). Although the latitude of the center of the positive GPH anomaly over the Korean Peninsula is relatively higher, i.e., by approximately 2°, than the observed result, the propagation of this wave toward both regions is similar to the observation (Figs. 2c, 7d).

## Summary and discussion

The large-scale atmospheric circulation patterns involved in Korean EHDs exhibit complex features in their temporal and spatial variations. To understand these features, many previous studies have used empirical methods, such as composite analysis or the empirical orthogonal function^5,12,14,18–22^. However, these empirical methods do not facilitate an understanding of the spatiotemporal variations associated with EHDs because the results analyzed via empirical methods depend on the analysis period (e.g., Fig. 1). For this reason, we divided the large-scale atmospheric circulation patterns associated with the EHDs in South Korea during JJA from 1982 to 2018 using an SOM. In addition, the FDR, which is a global significance test, was employed to determine the cluster number. As a result, three distinct SOM cluster patterns were identified (Fig. 2). EHDs in South Korea are modulated by two types of wave trains, as observed by previous studies^20,22^. Our clustering results roughly agree with the results of previous studies in terms of the two types of wave trains: C1 is associated with the eastward-propagating wave train, whereas C2 and C3 closely follow the northward-propagating wave train. However, there is a striking difference between C2 and C3 with respect to their spatial and temporal variations.

C1 is closely related to the eastward-propagating wave train. This wave propagates from northern Europe to East Asia across Eurasia and produces a positive GPH anomaly over the Korean Peninsula, inducing EHDs in South Korea (Fig. 4). C2 and C3 are influenced by the northward-propagating wave activated by enhanced convection over the subtropical WNP, developing favorable conditions for EHDs over South Korea. However, owing to differences in the features of enhanced convection over the subtropical WNP, the two clusters are distinct from each other. The large-scale atmospheric circulation pattern of C2 closely resembles the PJ teleconnection pattern (Fig. 5)^24^, whereas the teleconnection pattern in C3 is more complex. Based on the strong and eastward-extended enhanced convection of C3, as compared with that of C2, a positive GPH anomaly is generated over the Korean Peninsula and southern part of the Kamchatka peninsula (Fig. 6). Korean EHDs are directly related to the positive structure of the GPH anomaly over the Korean Peninsula and are reinforced by the GPH anomaly over the southern part of the Kamchatka Peninsula via atmospheric blocking (Fig. 6)^5^. Therefore, the mean temperature of C3 is higher than that of C2 (Table 1). The atmospheric circulation features of C2 and C3 were reproduced through LBM experiments from a wave propagation perspective (Fig. 7).

Again, the positive GPH anomaly over the Ural Mountains is a dominant feature of C3 (Figs. 2c, 6a), whereas the negative GPH anomaly in the same region consists of the eastward-propagating wave train in C1 (Figs. 2a, 4a). These significant anomalies may be canceled out when applying composite analysis (Fig. 1b–d), which can lead to a misinterpretation of the dynamic mechanism of EHDs in South Korea. To avoid this, clustering analysis was used in this study.

This study mainly focused on investigating the spatiotemporal variations and dynamic mechanisms of EHDs in South Korea at a daily timescale based on the results of the clustering method. However, at interannual and interdecadal timescales, the three SOM cluster patterns correlate significantly with the large-scale atmospheric and oceanic variability. For example, the interannual frequency of C1 strongly correlates with the Silk Road pattern^39^ (SRP) index (r = 0.55) from 1982 to 2018, where the SRP index is defined as the first PC of the 200-hPa meridional wind anomalies within 20°–60° N and 0–150° E. Previous studies have shown that the SRP has experienced an interdecadal phase transition from positive to negative since the late 1990s^40,41^. These observations can provide possible answers as to why the high-frequency years of C1 are concentrated before the late 1990s, which should be the focus of future research.

Finally, we revealed the three distinct atmospheric patterns associated with EHDs in South Korea using the SOM; nevertheless, the possibility of a methodological dependency of the current clustering results cannot be eliminated. To test this possibility, we classified the 850-hPa GPH anomaly fields involved in the Korean EHDs into three cluster patterns using the *K*-means clustering method. A cluster that related to the eastward-propagating wave train was well-classified by both clustering methods (Fig. 2a, Supplementary Fig. S6a). However, the spatial features of the remaining clusters varied considerably between the clustering methods. For example, the second cluster of the *K*-means clustering method can be interpreted as a mixture pattern of C2 and C3 of the SOM (Figs. 2b, c, Supplementary Fig. S6b) that would have resulted from the differences in the updated equation of the weight vector in the clustering methods. This implies that the clustered patterns and their physical interpretations associated with EHDs in South Korea may depend on the clustering method, and thus, require further analysis.

## Methods

### Observation and reanalysis datasets

This study utilized the dataset for the hourly observed temperatures obtained from 45 stations of the Korea Meteorological Administration (KMA) from 1982 to 2018 (https://data.kma.go.kr/cmmn/main.do). The observation stations are spread almost entirely over South Korea, except for the islands. To determine the daily maximum temperature representing South Korea, we calculated the spatial average of the hourly temperatures at the 45 stations and selected the maximum spatially averaged temperature.

To comprehend the dynamic mechanism between each cluster pattern for Korean EHDs and the large-scale variability, we used the daily mean GPH, horizontal wind, and 2-m temperature from the National Centers for Environmental Prediction–National Center for Atmospheric Research (NCEP–NCAR) reanalysis version 1^42^ (2.5° longitude × 2.5° latitude horizontal resolution) (https://psl.noaa.gov/data/gridded/data.ncep.reanalysis.html). The dataset for the daily mean OLR from the polar-orbiting satellites from the NOAA was also used^43^ (https://psl.noaa.gov/data/gridded/data.interp_OLR.html). The atmospheric variables used in this study are based on the anomaly after removing the daily climatological mean for the period from 1982 to 2018.

### Definition of EHD

Generally, the KMA defines a heat wave day as a period of at least two consecutive days whose daily maximum temperatures exceed 33 ℃^5,18–20^. If we apply this criterion on the daily maximum temperatures for JJA from 1982 to 2018, only 100 days can be defined as heat wave days. The purpose of this study is to classify the large-scale atmospheric circulation patterns associated with EHDs using a statistically based clustering method; however, a total of 100 heat wave days may not ensure statistical significance. Therefore, to obtain as much SOM input as possible, EHD (the number of days when the daily maximum temperature exceeds the 90th percentile threshold of the JJA daily maximum temperatures from 1982 to 2018^12,22^) was used to represent extreme high-temperature events. Consequently, the threshold was set to 31.50 ℃, which resulted in a total of 341 days identified as EHDs.

### SOM

The SOM^44,45^ is a type of artificial neural network analysis tool characterized by unsupervised learning; its networks learn to self-organize clusters from input data without external aid^33^. The SOM consists of two fully-connected layers, i.e., an arbitrary dimensional uninterrupted input layer and a one- or two-dimensional interrupted output layer. Through iterative training and mapping, an input vector is assigned to the output layer.

The first step of the training process is the random generation of *K* weight vectors, where the cluster number *K* is determined by the user. The next step of the training process is matching (or competition). This process calculates the Euclidean distances between the input vector and *K* weight vectors, and selects the weight vector with the minimum Euclidean distance as follows:

1 $$ {\text{Euclidean~distance}} = {\text{~}}\left| {X - W_{k} } \right| = \sqrt {\mathop \sum \limits_{{i = 1}}^{M} \left( {X_{i}  - W_{{ik}} } \right)^{2} } , $$

where *X* and *W*_*k*_ are the input and weight vectors of the *K*th cluster, respectively, and *M* is the total number of grid points corresponding to the input and weight vectors. The selected weight vector and its nearest neighbors within a user-defined neighborhood radius, which is determined through a specific topological neighborhood function, are updated as follows:

2 $$ W_{k} \left( {t + 1} \right) = W_{k} \left( t \right) + \alpha \left( t \right)nh\left( t \right)\left[ {X\left( t \right) - W_{k} \left( t \right)} \right], $$

where the learning rate, *α*, which is usually a monotonically decreasing function with a user-defined iteration number, *t*, was set to 0.05, and *nh* is the Gaussian neighborhood function. During the update, which is an essential part of the SOM procedure, each cluster is influenced by its neighboring clusters. Consequently, the SOM classification is considered to be nonlinear^28,33,45^, and the weight vectors are arrayed in an ordered listing so that the clusters can be easily diagnosed and interpreted^33^. As the conventional *K*-means clustering method does not contain the neighborhood function (i.e., the second term in Eq. (2) does not exist), the weight vector in this method was updated by the arithmetic mean of the input vectors in each cluster.

After 10 million iterations of the training process, the final weight vector was determined. Through the mapping process, each input vector was allotted a specific cluster based on the minimum Euclidean distance between the input and final weight vectors of the *K*^*th*^ cluster. The SOM-PAK version 3.4 used in this study can be downloaded for free from http://cis.legacy.ics.tkk.fi/hynde/lvq/.

The aim of this study is to classify the large-scale atmospheric circulation patterns of EHDs in South Korea. Previous studies have shown that teleconnection patterns are the most important large-scale variabilities modulating EHDs in South Korea^5,14,18–22^. The propagation mechanisms of these teleconnection patterns are expressed in GPH anomaly fields. Furthermore, the variabilities in the observed T_max_ and 850-hPa GPH fields over South Korea strongly correlate with each other on a daily to interannual timescale (not shown). Therefore, the daily mean 850-hPa GPH anomaly fields over the region, ranging from 10° S to 60° N and 0° to 180° E, attributed to EHDs in South Korea, were chosen as the input vectors for the SOM. We also tested the sensitivity of the SOM results based on their input regions. If we expanded/reduced the latitude and longitude by 5° and 10°, respectively, of the current SOM input region, the main conclusions were consistent with the current region.

### FDR

For the clustering analysis, the number of clusters must be specified prior to the clustering procedure. Although various useful heuristic methods for the selection of the number of clusters have been suggested in previous studies^46^, the optimal number of clusters remains elusive. Hence, we proposed the use of the FDR^47,48^, which is a global (i.e., field) significance test, for the determination of the maximum number of statistically discernible clusters. This method can be used to determine whether two cluster patterns (i.e., a pair of clusters) are statistically distinguishable from each other.

In the present application, the daily 850-hPa GPH anomaly fields over the region ranging from 10° S to 60° N and 0° to 180° E were used as the input vector for clustering using the SOM. They included 2117 grid points owing to their horizontal resolution. To determine whether the SOM cluster pattern, *i*, is statistically distinguishable from the SOM cluster pattern, *j*, we first calculated the *t* value using the two-tailed Student’s *t*-test for different means at each grid point (see Eqs. (3) and (4)). The null hypothesis states that the local (i.e., each grid point) composite of the 850-hPa GPH anomaly fields is the same in both clusters:

3 $$ t\left( {lon,~lat} \right) = \frac{{\overline{{GPH_{i} }} \left( {lon,lat} \right) - \overline{{GPH_{j} }} \left( {lon,lat} \right)}}{{S\left( {lon,lat} \right)\sqrt {\frac{1}{{n_{i} }} + \frac{1}{{n_{j} }}} }}, $$

where

4 $$ s\left( {lon,lat} \right) = ~\sqrt {\frac{{\left( {n_{i}  - 1} \right)S_{i} \left( {lon,lat} \right)^{2}  + \left( {n_{j}  - 1} \right)S_{j} \left( {lon,lat} \right)^{2} }}{{n_{i}  + n_{j} }}} . $$

The variables $\overline{{GPH_{i} }}$ ($\overline{{GPH_{j} }}$) and *S*_*i*_ (*S*_*j*_) denote the mean and standard deviations of the 850-hPa GPH anomaly fields for the SOM cluster pattern, *i* (*j*), respectively, and *n*_*i*_ (*n*_*j*_) is the sample size of the SOM cluster pattern, *i* (*j*).

After calculating the *t* values based on the *t*-test for each grid point, we converted the *t* values into *p* values and executed the FDR as follows:

5 $$ P_{{FDR}}  = \mathop {\max }\limits_{{1 \le m \le M}} \left[ {~p\left( m \right):p\left( m \right) \le q\cdot\frac{m}{M}} \right], $$

where *p*(*m*) denotes the *p* values that are sorted in ascending order and *m* varies from 1 to *M*, where *M* is the total number of grid points. The sorted *p* values were compared with *q*·*m/M*, where the global significance test level, *q*, was set to 0.01. If *q* is used in Eq. (5) instead of *q*·*m/M*, the test becomes a sequential local *t*-test. Using *q*·*m/M*, where *m/M* varies from approximately 0 to 1, the test becomes more stringent^30^. Therefore, *P*_*FDR*_ represents the probability that the corresponding null hypothesis, which is actually true, is wrongly rejected. If none of the grid points satisfies the condition shown in Eq. (5) for a pair of clusters, both clusters are statistically indistinguishable. In contrast, if at least one grid point satisfies the above-mentioned condition for a pair of clusters, the null hypothesis is rejected (i.e., the cluster pair is statistically distinguishable).

In summary, if all pairs of clusters are statistically distinguishable from each other when the number of clusters is *K* while at least one pair of clusters is statistically indistinguishable from each other when the number of clusters is *K* + 1, *K* is selected as the maximum number of statistically manifested clusters.

### LBM

To verify that the changes in the atmospheric response to the prescribed forcing (i.e., diabatic heating) are related to the clustered patterns, LBM^49^ experiments were conducted. The LBM is based on a dynamical core of version 5.4 g of the atmospheric general circulation model, cooperatively developed by the Center for Climate System Research and National Institute for Environmental Studies (from the user’s guide for the LBM package, which is freely available at https://ccsr.aori.u-tokyo.ac.jp/~lbm/lbm/doc2.2.pdf). This model is governed by primitive equations exactly linearized with respect to a basic state, which is horizontally represented by spherical harmonics with a resolution of T42 and vertically discretized using a finite difference to 20 sigma levels from 0.995 to 0.008.

## Supplementary information

Supplementary Information.
