# Supplementary material for: Three distinct atmospheric circulation patterns associated with high temperature extremes in South Korea
Source: Sci Rep. 2021 Jun 18;11:12911. doi: 10.1038/s41598-021-92368-9 (PMC8213777; doi:10.1038/s41598-021-92368-9)
Supplement: Supplementary file 1 — Supplementary Information. [file 41598_2021_92368_MOESM1_ESM.pdf]

Supplementary information for

Three distinct atmospheric circulation patterns associated with high temperature extremes in South Korea

Han-Kyoung Kim[1], Byung-Kwon Moon[1*], Maeng-Ki Kim[2],

Jong-Yeon Park[3], and Yu-Kyung Hyun[4]

[1]Division of Science Education/Institute of Fusion Science, Jeonbuk National University, Jeonju, South Korea

[2]Department of Atmospheric Science, Kongju National University, Gongju, South Korea

[3]Department of Earth and Environmental Sciences, Jeonbuk National University, Jeonju, South Korea

[4]Operational Systems Development Department, National Institute of Meteorological Science, South Korea

*Corresponding author:
Dr. Byung-Kwon Moon
Tel: +82-63-270-2824
Fax: +82-63-270-2802
Email: moonbk@jbnu.ac.kr

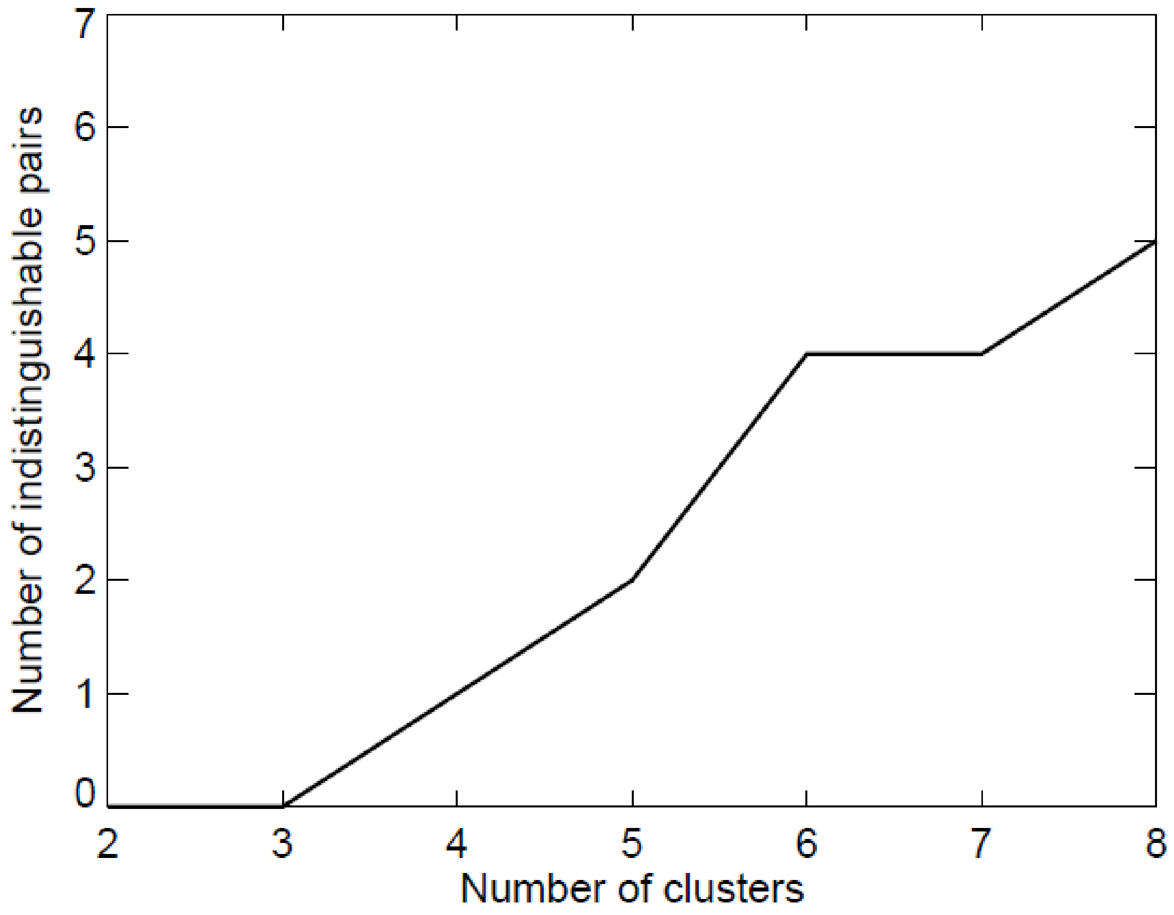

Figure S1. Number of SOM cluster pairs that are statistically indistinguishable at the 99% confidence level as a function of the number of SOM clusters.

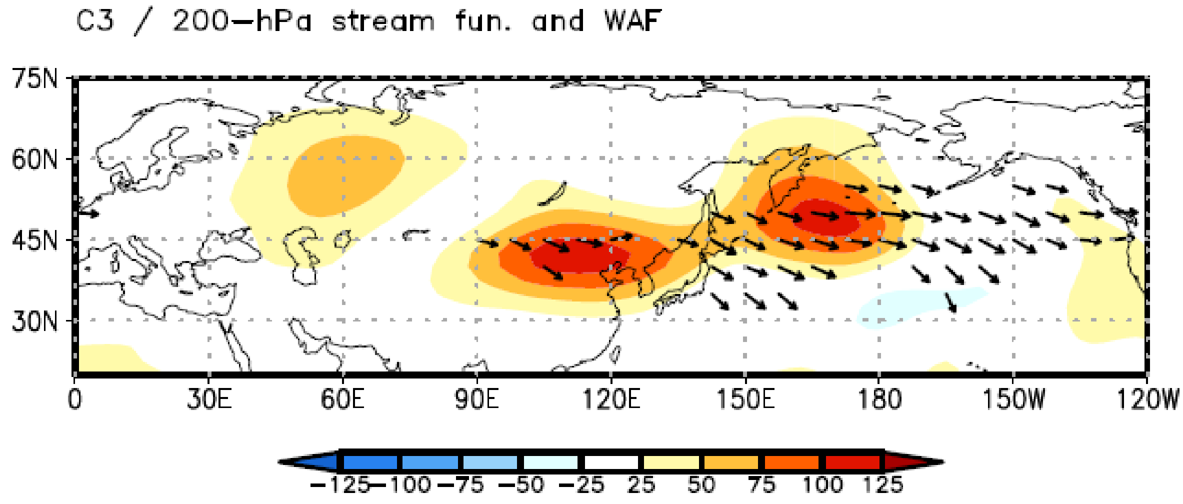

Figure S2. Composite map of the 200-hPa stream function (shaded; $m^2\ s^{-1}$) and WAF (vector; $m^2\ s^{-2}$) for C3. WAF was omitted when its magnitude was less than 15 $m^2\ s^{-2}$. Map is generated using GrADS version 2.1.a1 (http://cola.gmu.edu/grads/downloads.php).

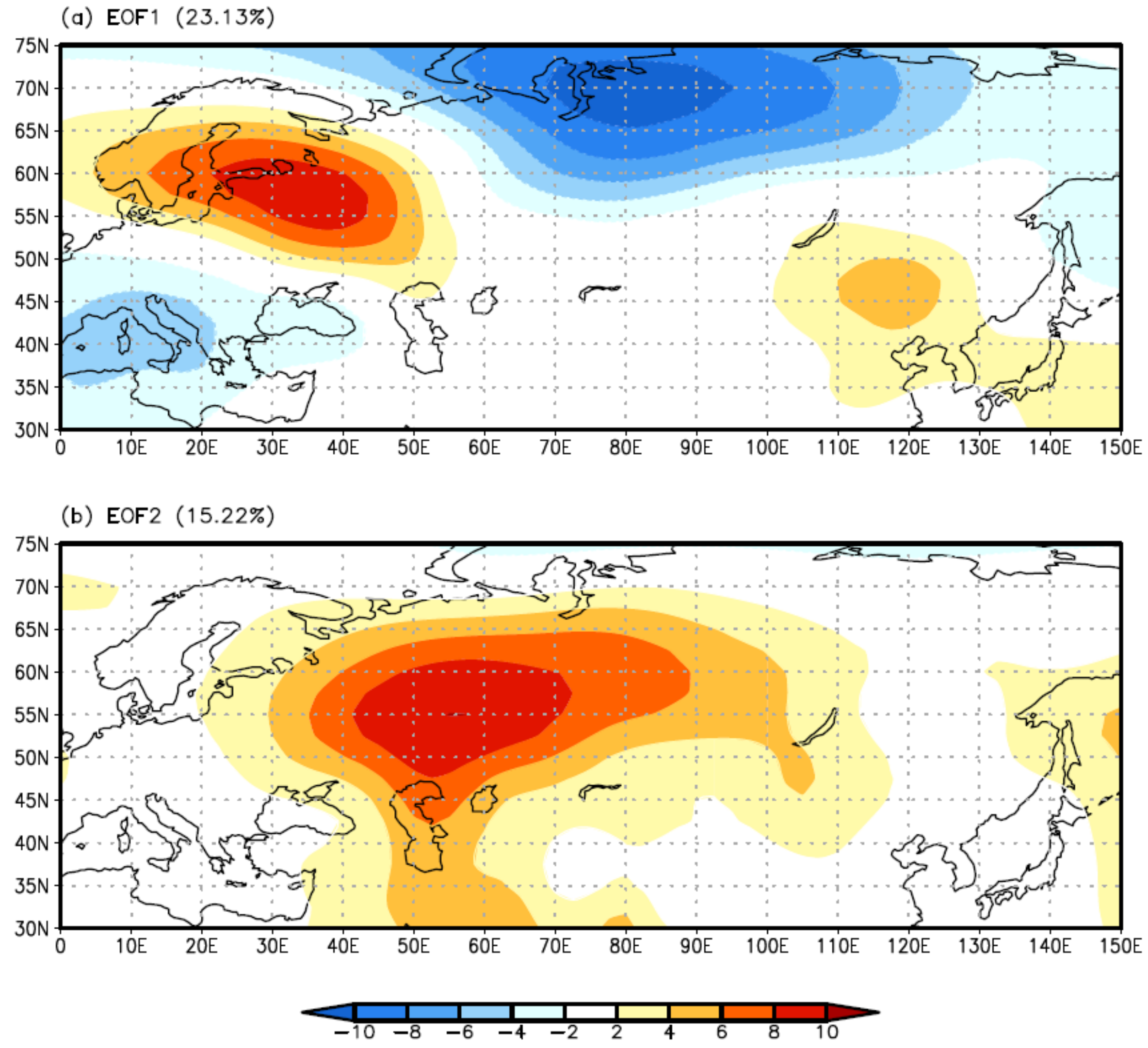

Figure S3. Spatial patterns of the (a) first and (b) second leading EOF mode of the JJA mean 850-hPa GPH (shaded; m) anomaly over the Eurasian continent. The percentages of explained variance to the total variance are shown in parentheses. Maps are generated using GrADS version 2.1.a1 (http://cola.gmu.edu/grads/downloads.php).

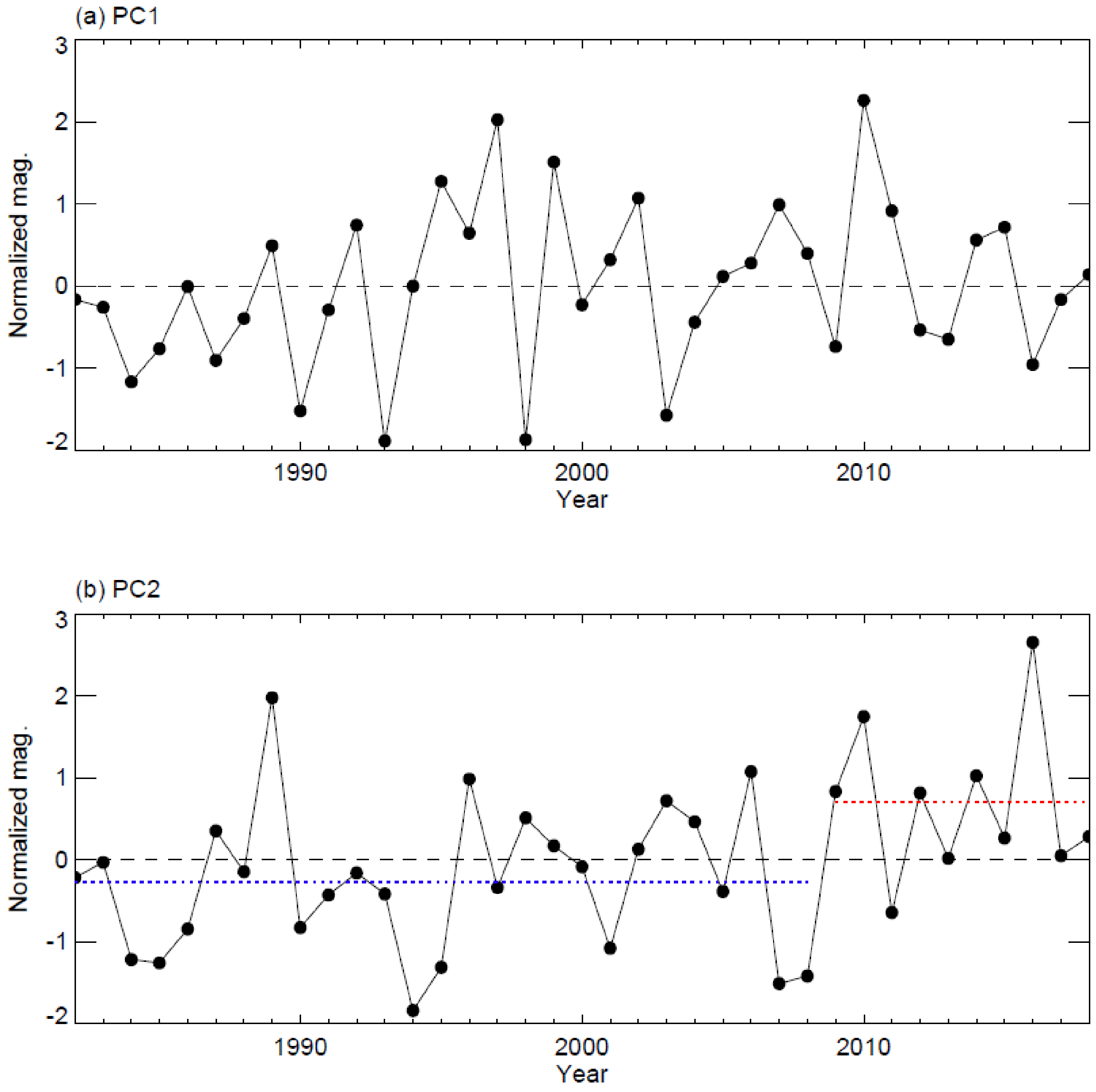

Figure S4. Normalized time series of the (a) first and (b) second PC of the JJA mean 850-hPa GPH anomaly over the Eurasian continent. The blue (red) dotted horizontal line in (b) denotes the mean normalized magnitude of P1 (P2). The Rodionov regime shift algorithm is applied to detect the interdecadal change point.

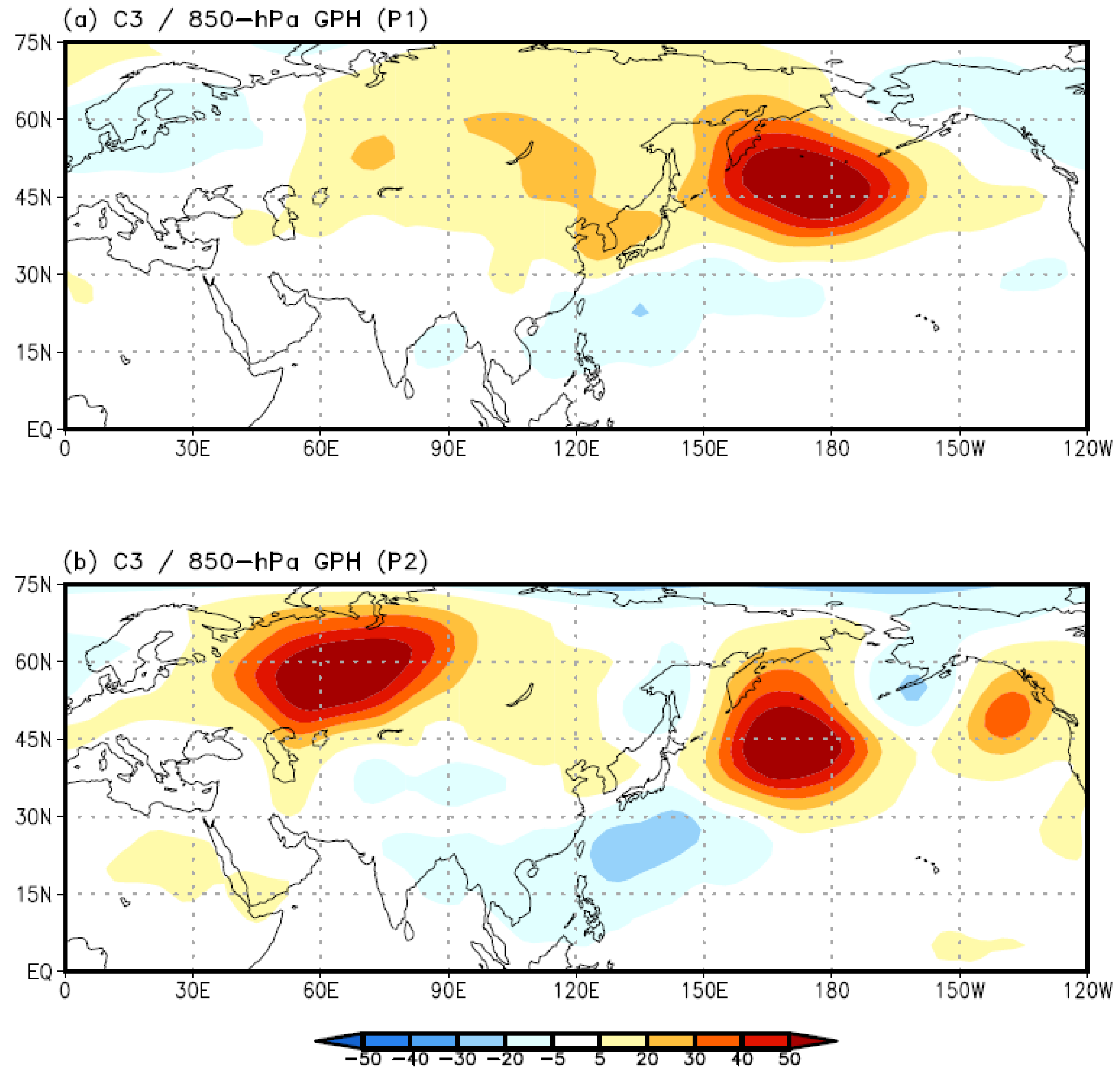

Figure S5. Composite map of the 850-hPa GPH (shaded; m) anomalies for C3 for the period of (a) P1 and (b) P2. Maps are generated using GrADS version 2.1.a1 (http://cola.gmu.edu/grads/downloads.php).

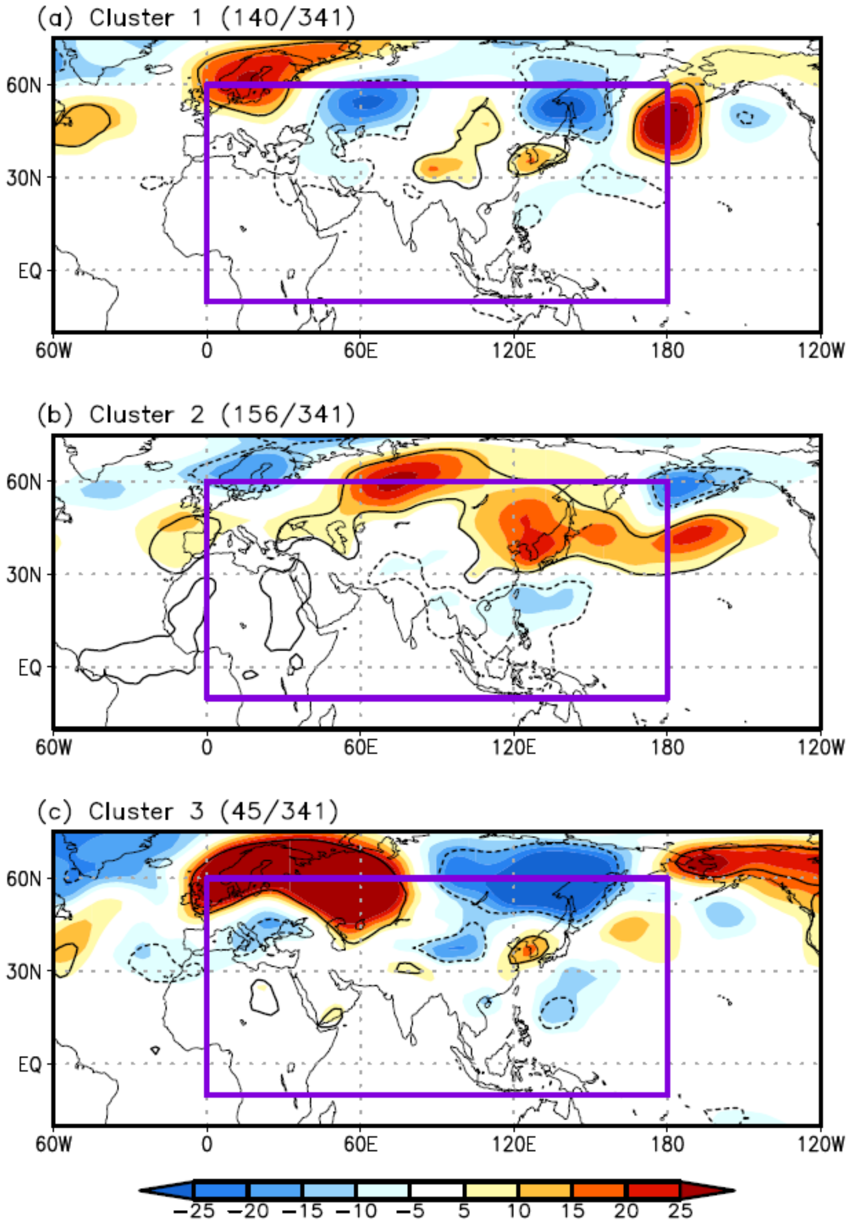

Figure S6. Composite maps of the 850-hPa GPH (shaded; m) anomalies for the three *K*-means cluster patterns. The purple box (i.e., 0–180° E and 10° S–60° N) denotes the region of the input vector for the SOM. Contours represent statistically significant areas satisfying the 99 % confidence level based on the Student's *t*-test. The number of EHDs in each cluster are shown in parentheses. Maps were generated using GrADS version 2.1.a1 (http://cola.gmu.edu/grads/downloads.php).
